# Supplementary material for: Single-cell multiomics data integration and generation with scPairing
Source: Cell Rep Methods. 2025 Oct 27;5(11):101211. doi: 10.1016/j.crmeth.2025.101211 (PMC12664900; doi:10.1016/j.crmeth.2025.101211)
Supplement: Document S1. Figures S1–S7, Tables S1–S6, and Note S1 [file mmc1.pdf]

Cell Reports Methods, Volume *5*

Supplemental information

Single-cell multiomics data integration and generation with scPairing

Jeffrey Niu, Carlos Vasquez-Rios, and Jiarui Ding

Supplementary Figures

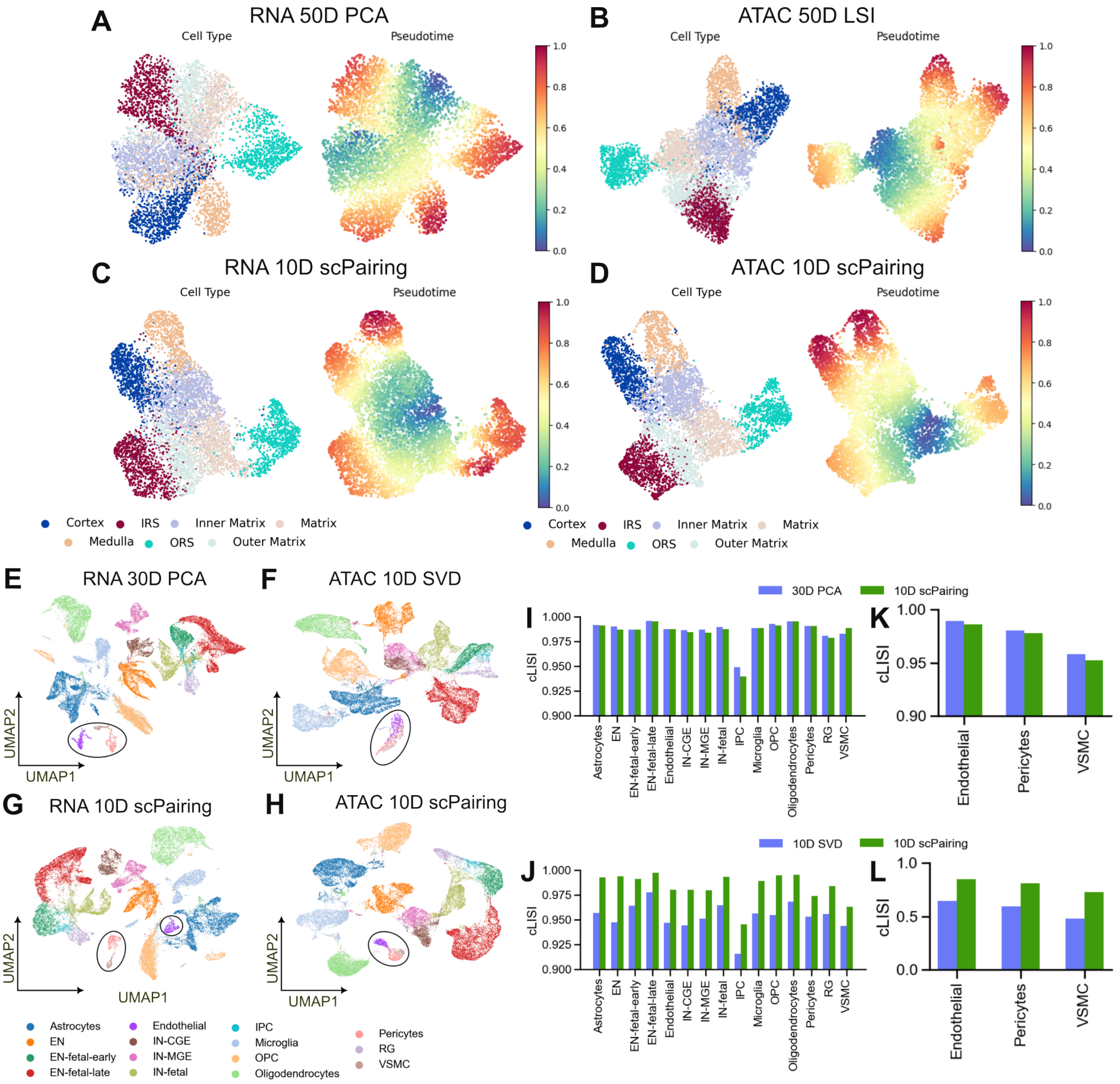

Figure S1: Alignment of modalities transfers modality-specific information onto both modalities, related to Fig. 2. (A–D) uniform manifold approximation and projection (UMAP) visualization of cell type and pseudotime of mouse skin data using single-cell RNA sequencing (scRNA-seq) principal component analysis (PCA) embeddings (A), single-cell assay for transposase-accessible chromatin with sequencing (scATAC-seq) latent semantic indexing (LSI) embeddings (B), scPairing's transformation of the PCA embeddings (C), and scPairing's transformation of the LSI embeddings (D). (E–L) Alignment of human cerebral cortex multiomics data. (E and F) UMAP visualization of cell types following the data processing procedure from Zhu *et al.*,[1] with the 30-dimension PCA embeddings (E) and the 10-dimension SVD embeddings (F). (G and H) UMAP visualization of cell types after applying scPairing to the PCA and SVD embeddings from (E) and (F). The three blood vessel subtypes are highlighted in the black circle. (I and J) Comparison of cell type Local Inverse Simpson's Index (LISI) (cLISI) between the PCA embeddings and scPairing embeddings in the scRNA-seq modality (I), and between the SVD embeddings and scPairing embeddings in the scATAC-seq modality (J). (K and L) Comparison of cLISI computed only on the three blood vessel cell subtypes. (K) compares the cLISI between the PCA embeddings and scPairing embeddings in the scRNA-seq modality. (L) compares the cLISI between the SVD embeddings and scPairing embeddings in the scATAC-seq modality.

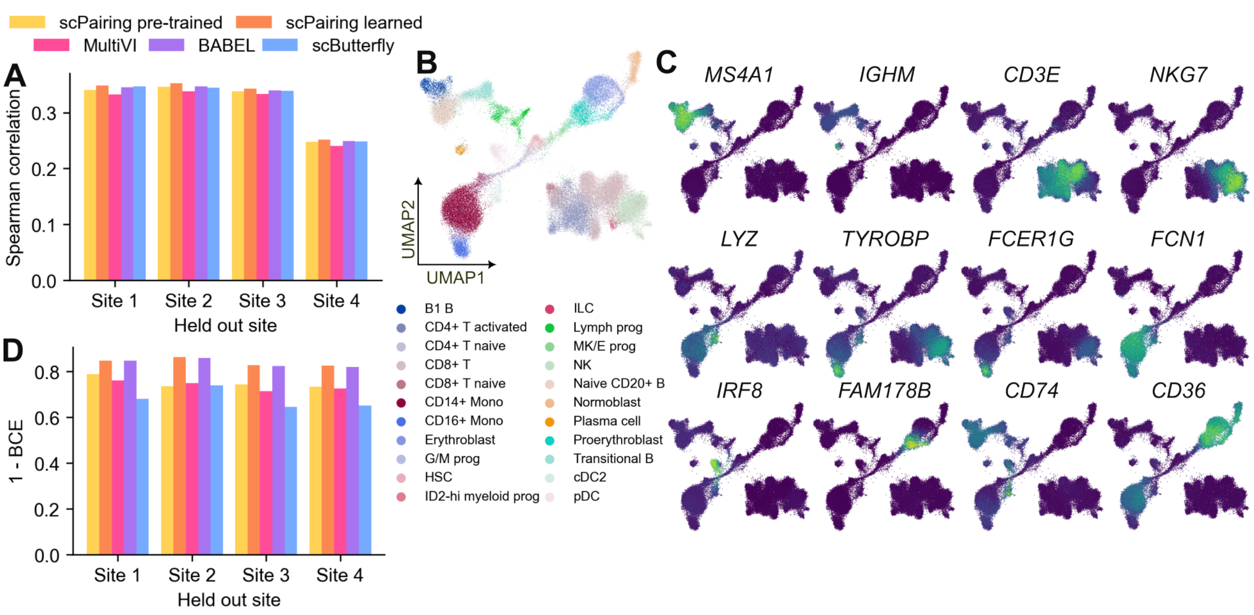

Figure S2: Benchmarking cross-modal imputation using pre-trained and learned decoders, related to Fig. 2. (A) Comparison of cross-site scATAC-seq to scRNA-seq imputation with Spearman correlations between imputed and true gene counts. (B) UMAP visualization of the scRNA-seq bone marrow mononuclear cell (BMMC) benchmarking dataset using scVI embeddings. (C) Visualization of scPairing marker gene expression imputations. We applied scVI and PeakVI pre-trained decoders to impute the counts. (D) Comparison of cross-site scRNA-seq to scATAC-seq imputation with binary cross-entropy between the imputed and true binarized peaks.

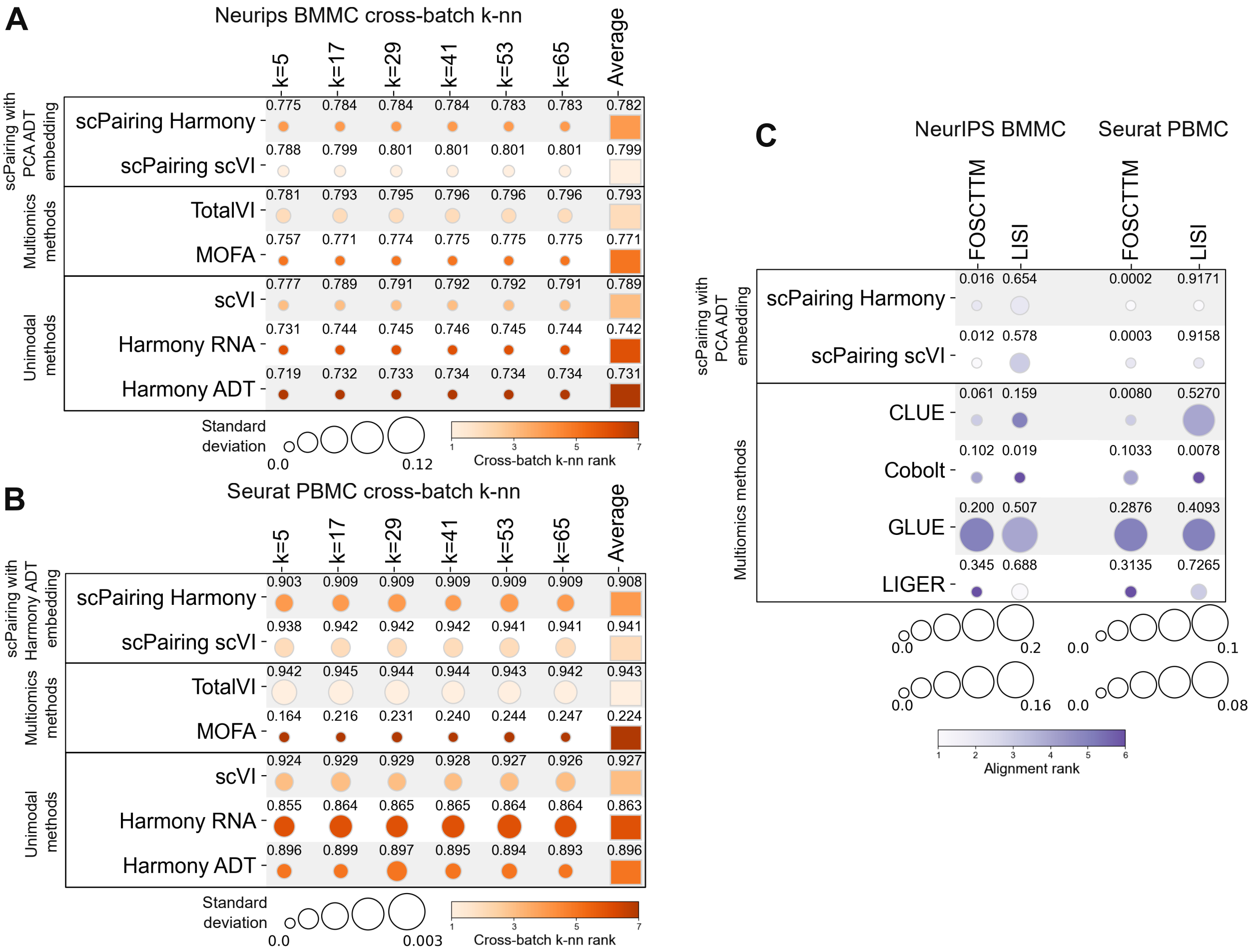

Figure S3: Benchmarking integration of CITE-seq data, related to Fig. 2. (A and B) Comparison of biological structure preservation in the BMMC data (A) and the peripheral blood mononuclear cell (PBMC) data (B) quantified by k-nearest neighbor cell type classification accuracies, where we predicted cell types for one batch given the cells from the remaining batches (cross-batch k-nn) or predicted cell types for held-out cells using 10-fold cross-validation (10-fold k-nn). (C) Comparison of modality alignment performance with Fraction of Samples Closer than True Match (FOSCTTM) and LISI computed across all cells in the BMMC data (left two columns) and the PBMC data (right two columns). scPairing Harmony and scPairing scVI refers to scPairing with Harmony-corrected PCA and scVI used as scRNA-seq embedding, respectively. All experiments were repeated for five trials. The mean of each metric is labeled and the standard deviation is given by the circle size.

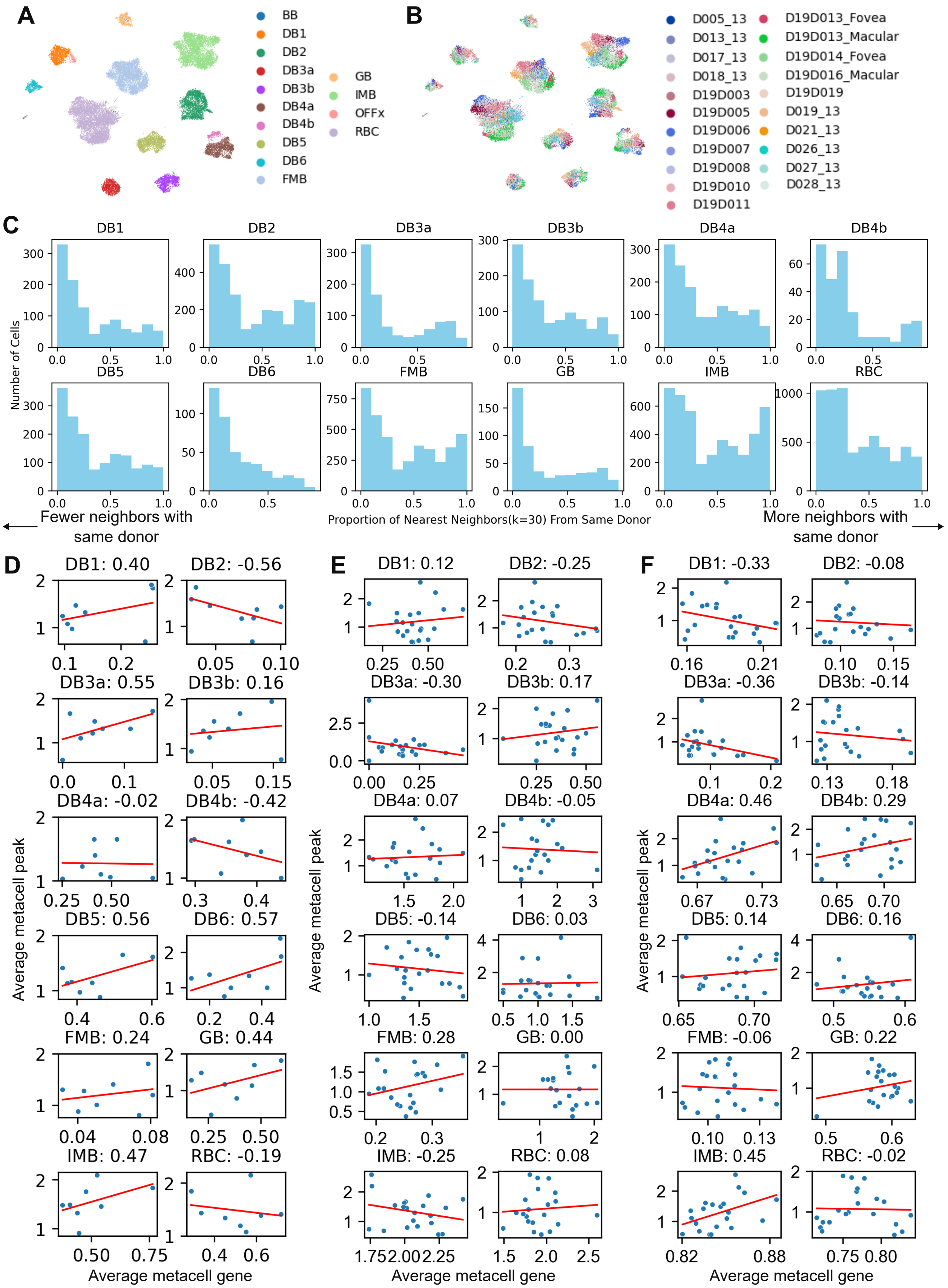

A
BB
DB1
DB2
DB3a
DB3b
DB4a
DB4b
DB5
DB6
FMB
GB
IMB
OFFx
RBC
B
D005_13
D013_13
D017_13
D018_13
D19D003
D19D005
D19D006
D19D007
D19D008
D19D010
D19D011
D19D013_Fovea
D19D013_Macular
D19D014_Fovea
D19D016_Macular
D19D019
D019_13
D021_13
D026_13
D027_13
D028_13
C
Number of Cells
Fewer neighbors with same donor
Proportion of Nearest Neighbors(k=30) From Same Donor
More neighbors with same donor
D
DB1: 0.40
DB2: -0.56
DB3a: 0.55
DB3b: 0.16
DB4a: -0.02
DB4b: -0.42
DB5: 0.56
DB6: 0.57
FMB: 0.24
GB: 0.44
IMB: 0.47
RBC: -0.19
Average metacell peak
Average metacell gene
E
DB1: 0.12
DB2: -0.25
DB3a: -0.30
DB3b: 0.17
DB4a: 0.07
DB4b: -0.05
DB5: -0.14
DB6: 0.03
FMB: 0.28
GB: 0.00
IMB: -0.25
RBC: 0.08
Average metacell peak
Average metacell gene
F
DB1: -0.33
DB2: -0.08
DB3a: -0.36
DB3b: -0.14
DB4a: 0.46
DB4b: 0.29
DB5: 0.14
DB6: 0.16
FMB: -0.06
GB: 0.22
IMB: 0.45
RBC: -0.02
Average metacell peak
Average metacell gene

Figure S4: Donor-aggregated metacells in retina data, related to Fig. 3 and Methods. (A and B) UMAP visualization of bipolar retina cells from Wang *et al.*[2] using LSI dimensionality reduction without batch correction, colored by cell subtype (A) and donor (B). (C) Proportion of nearest neighbors ($k = 30$) having the same donor for each cell subtype. (D) Correlations computed on the Wang *et al.* paired multiomics dataset using donor-aggregated metacells. The data contained four donors with two retina each, for eight batches in total. (E) Correlations computed on the artificial pairings using donor-aggregated metacells. The data contained 21 donors. (F) Correlations computed on the gene activities and canonical correlation analysis (CCA) imputations using donor-aggregated metacells. This is the same data as in (E), with 21 donors.

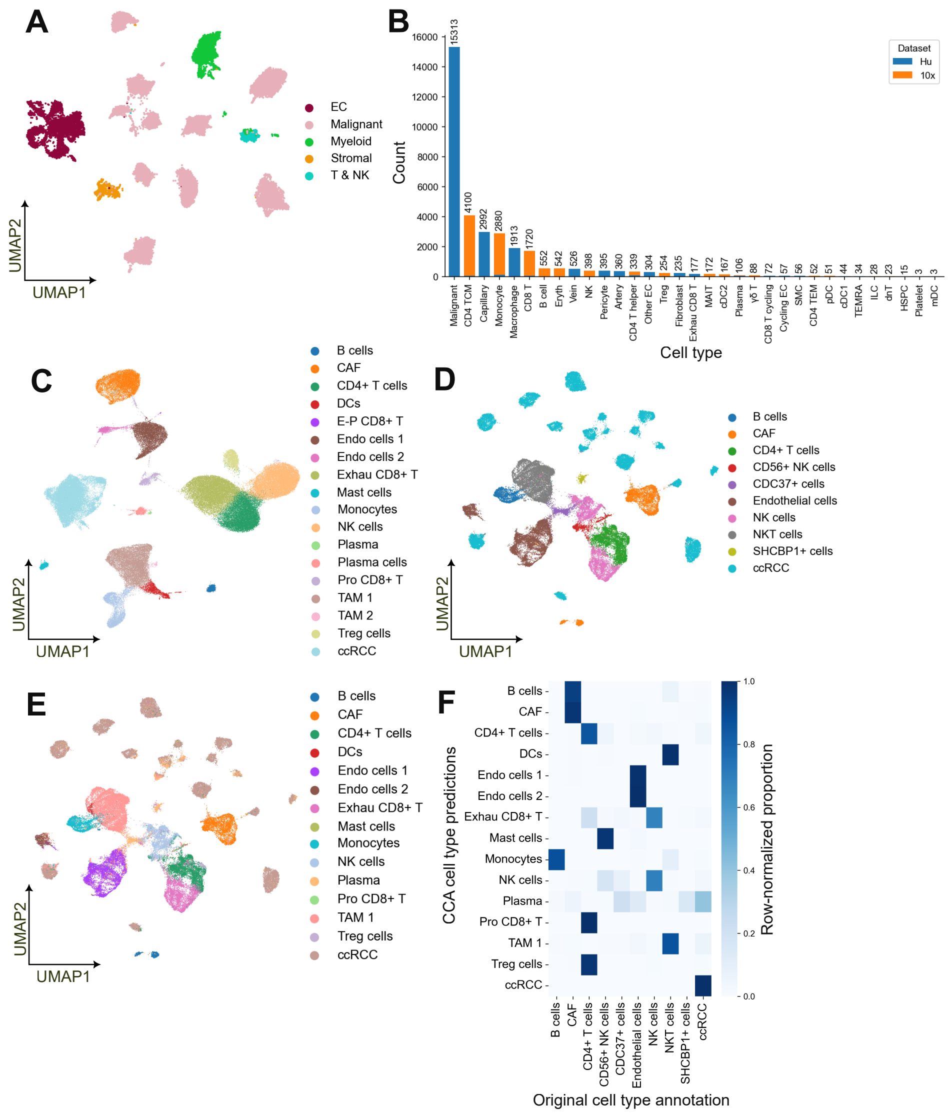

Figure S5: Cell type annotations of clear cell renal cell carcinoma (ccRCC) bridge and single-modality data, related to Fig. 6. (A) UMAP visualization of cell types in the Hu *et al.* data.[3] (B) Cell type counts in the bridge data after adding PBMCs from 10x Multiome.[4] (C) Original cell type annotations of ccRCC scRNA-seq data from Yu *et al.*,[5] whose annotations were transferred onto the scATAC-seq data in (D and E). (D) Original cell type annotations of the ccRCC scATAC-seq data from Yu *et al.*, which were annotated on the basis of gene scores, differentially expressed peaks, and transcription factor analysis.[5] (E) Cell type annotations of the ccRCC scATAC-seq data in (D) following label transfer using CCA. (F) Comparison of CCA cell type annotations of the scATAC-seq data in (C) against the original annotations in (D). The heatmap is normalized per row.

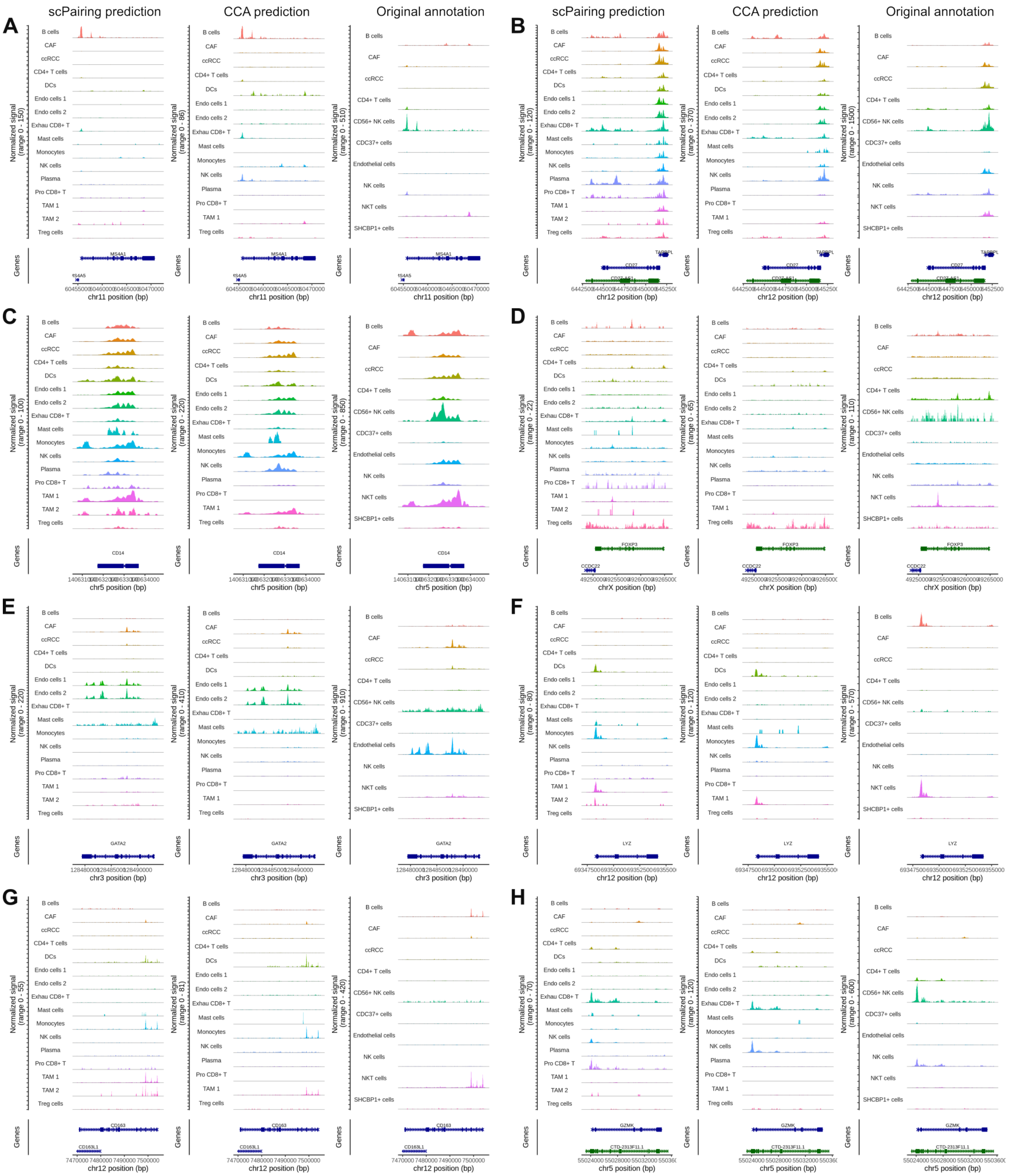

Figure S6: Accessibility profiles of ccRCC scATAC-seq profiles at marker genes, related to Fig. 6. For each gene, the accessibilities are grouped by cell type annotations from scPairing (left column), CCA (middle column), and the original annotations (right column). (A–H) Accessibility plots for *MS4A1* (A, a B cell marker), *CD27* (B, plasma cell marker), *CD14* (C, expressed in monocytes), *FOXP3* (D, a regulatory T cell marker), *GATA2* (E, mast cells), *LYZ* (F, monocytes/macrophages/dendritic cells), *CD163* (G, monocytes/macrophages/dendritic cells), and *GZMK* (H, exhausted CD8^{+} T cells). Accessibility for E-P CD8+ T cells are omitted due to low cell number.

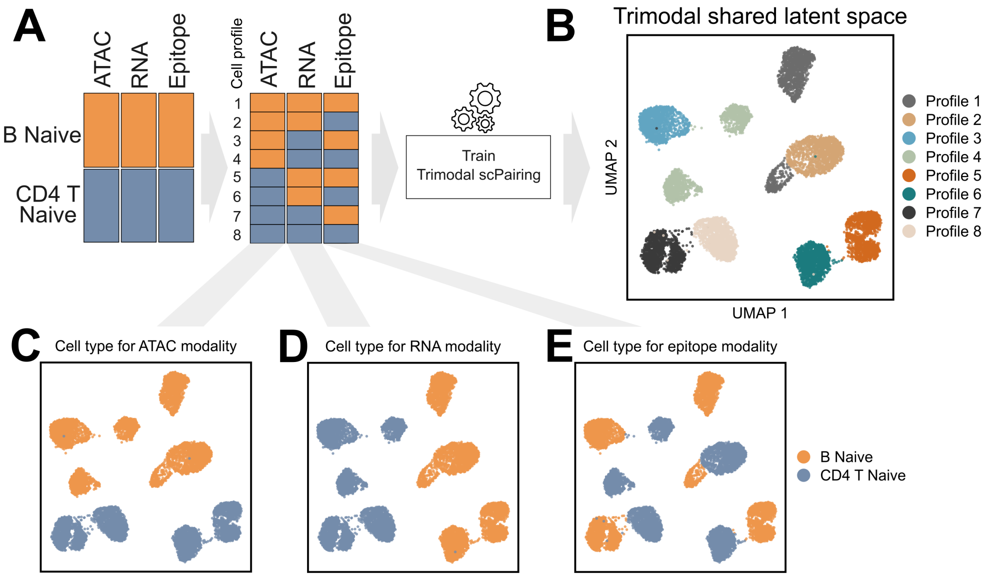

Figure S7: scPairing separates cells with shuffled modalities, related to Fig. 7. (A) Schematic of the modality shuffling, with eight profiles generated from all combinations of scATAC-seq, scRNA-seq, and epitope modalities of B naive and CD4 T naive cells. (B) UMAP visualization of scPairing embeddings of the eight shuffled profiles. (C–E) UMAP visualization of the individual modalities.

Supplementary notes

S1 Trimodal pairing algorithm, related to Methods

Algorithm 1 Trimodal pairing

Require: $A \in [0,1]^{n \times m}, B \in [0,1]^{n \times l}, C \in [0,1]^{m \times l}$, A, B, C are pairwise cosine similarity matrices between RNA and ATAC, RNA and epitope, and ATAC and epitope, respectively. There are n RNA profiles, m ATAC profiles, and l epitope profiles.

Require: cutoff $\in [0,1]$

Ensure: A maximal trimodal pairing

Without loss of generality, assume that RNA has the fewest number of profiles
Pairings $\leftarrow \{\}$
All RNA, ATAC, and epitope profiles are in consideration
while $\exists$ RNA profiles for consideration **do**
    $i \leftarrow$ random RNA profile
    $j, k \leftarrow$ ATAC and epitope profiles that maximize average similarity between i, j, k
    if average similarity of $i, j, k >$ cutoff **then**
        Pairings $\leftarrow$ Pairings $\cup \{(i, j, k)\}$
        Remove i, j, k from consideration
    else
        Remove i from consideration
    end if
end while
return Pairings

Supplementary tables

Number of cells	CPU runtime (s)	GPU runtime (s)
5000	260	11.2
	275	11.4
	285	11.8
10000	575	23.0
	626	23.2
	663	23.6
20000	1279	45.0
	1277	46.3
	1274	45.0
40000	1395	93
	1366	90
	1429	92
69249	2909	139
	2686	137
	2725	138

Table S1: Runtime of scPairing, related to Fig. 2. Runtime using a single Intel Xeon Gold 6130 CPU or a single NVIDIA Tesla V100 GPU in seconds across five dataset sizes. Each dataset size was run for three trials. The result of each trial is reported.

MD loss	BD loss	CS loss	Test BMMC data FOSCTTM	Test BMMC data Proportion of cells paired	Retina data Proportion of cells paired
✓	✓	✓	0.010442 ± 0.000151	0.991370 ± 0.000695	0.845802 ± 0.015269
✓	✓	✗	0.010712 ± 0.000213	0.943339 ± 0.004580	0.404926 ± 0.128817
✓	✗	✓	0.010481 ± 0.000112	0.992948 ± 0.000384	0.762668 ± 0.004222
✓	✗	✗	0.010654 ± 0.000120	0.946065 ± 0.002797	0.272155 ± 0.076137
✗	✓	✓	0.010582 ± 0.000322	0.990906 ± 0.000549	0.808498 ± 0.015745
✗	✓	✗	0.010769 ± 0.000327	0.935046 ± 0.003350	0.299533 ± 0.098023
✗	✗	✓	0.010537 ± 0.000187	0.993273 ± 0.000207	0.769556 ± 0.004856
✗	✗	✗	0.010660 ± 0.000131	0.945184 ± 0.001766	0.348049 ± 0.075139

Table S2: Average FOSCTTM and proportion of cells paired on eight variations of scPairing losses, related to Fig. 4. Each of the eight combinations of modality discriminative (MD) loss, batch discriminative (BD) loss, and cosine similarity (CS) loss were tested. The FOSCTTMs and proportion of cells paired were calculated from the test data after applying each scPairing variant. The proportion of cells paired is the number of pairings produced by the linear sum assignment algorithm divided by the total number of cells in the test BMMC data, or the number of pairings divided by the total number of single-nuclei ATAC-seq (snATAC-seq) cells in the retina data. Each scPairing variant was evaluated across five trials, with the mean and standard error reported.

ε used in pairing	BMMC test data proportion of cells paired	Retina data proportion of cells paired
0.5	1.0 ± 0.0	1.0 ± 0.0
0.4	1.0 ± 0.0	1.0 ± 0.0
0.3	1.0 ± 0.0	1.0 ± 0.0
0.2	1.0 ± 0.0	$0.99998824 \pm 5.73 \times 10^{-6}$
0.1	$0.99993041 \pm 1.03 \times 10^{-5}$	$0.99897859 \pm 3.48 \times 10^{-4}$
0.05	$0.99137041 \pm 6.22 \times 10^{-4}$	$0.98290358 \pm 3.57 \times 10^{-3}$
0.02	$0.82583077 \pm 5.97 \times 10^{-3}$	0.84580158 ± 0.01365
0.01	$0.43916952 \pm 9.95 \times 10^{-3}$	0.60889872 ± 0.01877

Table S3: Average proportion of cells paired when varying ε, related to Fig. 4. The proportion of cells paired is the number of pairings produced by the linear sum assignment algorithm divided by the total number of cells in the case of the BMMC test data, or the total number of scATAC-seq cells in the retina data. Each scPairing variant was evaluated across five trials, with the mean and standard error reported.

R-DC-like scRNA-seq cell barcode	Artificially paired scATAC-seq cell barcode	Successful pairing	True match
TGCCGGTAGGCATTAC-1	TGCCGGTAGGCATTAC-1	✓	✓
TAGTGTGGTACGGTAC-1	AACCCGCAGCAACATC-1	✓	✗
AAGCCTTAGTGAGAGC-1	GTTACCTGTTAGCCAA-1	✗	✗
CTTACTAGTGCTCCGT-1	GTATTGCAGCTTCTCA-1	✓	✗
GTACCGGGTCCTTCAG-1	TCCATCATCCCGAAGC-1	✓	✗
TCCTTTACAGCAACAG-1	TCCTTTACAGCAACAG-1	✓	✓
ACTTTGTTCGCTAGAT-1	ACTTTGTTCGCTAGAT-1	✓	✓
TCCATCATCCCGAAGC-1	AAGCCTTAGTGAGAGC-1	✓	✗
GATTGCAGTCCAAGAC-1	GATTGCAGTCCAAGAC-1	✓	✓
GCCAATAGTGATGAAA-1	ACGGGAAGTATTGGTG-1	✓	✗
GTATTGCAGCTTCTCA-1	GTACCGGGTCCTTCAG-1	✓	✗
TCTTTGTAGTTCCCAC-1	TCACTGACATAAGCAA-1	✗	✗
AACCCGCAGCAACATC-1	TAGTGTGGTACGGTAC-1	✓	✗
ATGCAGGCATTGTTGG-1	ATGCAGGCATTGTTGG-1	✓	✓
CGCTTAACACCAAAGG-1	CGCTTAACACCAAAGG-1	✓	✓
TTCCTTGAGTAATCCA-1	GTCTTTAGTTGCTGGG-1	✗	✗
CCAGGATGTTTATCTG-1	CCAGGATGTTTATCTG-1	✓	✓
ACCAGGACATAATCAC-1	ACCAGGACATAATCAC-1	✓	✓
ATATAGGCATTAGCCA-1	CGTGACATCAGGGCCT-1	✓	✗
CCGGTAGGTAGCAGCT-1	CCGGTAGGTAGCAGCT-1	✓	✓
CTACAACAGGAAACTG-1	TCTTTGTAGTTCCCAC-1	✓	✗
ACGGGAAGTATTGGTG-1	ATATAGGCATTAGCCA-1	✓	✗
CACTTAAAGGAACACA-1	CACTTAAAGGAACACA-1	✓	✓
CGTGACATCAGGGCCT-1	CTACAACAGGAAACTG-1	✓	✗
GGTCCTGCAACCTAAT-1	GGTCCTGCAACCTAAT-1	✓	✓
GATGCGACAGGATAAC-1	CTTACTAGTGCTCCGT-1	✓	✗
GCTGAGGAGATAAAGC-1	GCTGAGGAGATAAAGC-1	✓	✓

Table S4: Re-pairings of human tonsil R-DC-like cells, related to Fig. 5. The cells in the first column were identified as likely R-DC-like cells from their gene expression profiles. The second column indicates the scATAC-seq profile that was paired with each R-DC-like gene expression profile. The third column indicates whether the scRNA-seq and scATAC-seq profiles both belonged to R-DC-like cells. The fourth column indicates whether the scRNA-seq and scATAC-seq profiles came from the same cell, meaning scPairing recapitulated the true pairing of the two modalities. The cell barcodes listed have had their prefixes stripped off, but no duplicate barcodes are present in the R-DC-like cells.

R-DC-like scRNA-seq cell barcode	Artificially paired scATAC-seq cell barcode	Successful pairing	True match
AGCCTGGGTCCGTAGG-1	CATCCTCAGTGCTGTG-1	✓	✗
ACGAATCTCCGCAAGC-1	AGCCTGGGTCCGTAGG-1	✓	✗
AGTGAGTCAGCTTAAT-1	AGTGAGTCAGCTTAAT-1	✓	✓
GATGCAGCAGTCTATG-1	TGTAACTCAGAGGGAG-1	✓	✗
GTGCACGGTTAAGCCA-1	TACTAAGTCGCTTGCT-1	✗	✗
GGTGCTGGTCAAGTAT-1	TGACTTAAGTATTGCA-1	✓	✗
TGTAAGTGTTTAGTCC-1	GATGCAGCAGTCTATG-1	✓	✗
GATGAGGGTAAGTCGC-1	GGTGCTGGTCAAGTAT-1	✓	✗
TGACTTAAGTATTGCA-1	TGAGTTTCACCATATG-1	✗	✗
GTCTAATCATGTTGCA-1	GTCTAATCATGTTGCA-1	✓	✓
TGTAACTCAGAGGGAG-1	TAAGCCAGTGCGCAAT-1	✗	✗
CATCCTCAGTGCTGTG-1	ACGAATCTCCGCAAGC-1	✓	✗
TAGCGGACAGACAAAC-1	TAGCGGACAGACAAAC-1	✓	✓

Table S5: Re-pairings of intestine R-DC-like cells, related to Fig. 5. The cells in the first column were identified as likely R-DC-like cells from their gene expression profile. The second column indicates the scATAC-seq profile that was paired with each R-DC-like gene expression profile. The third column indicates whether the scRNA-seq and scATAC-seq profiles both belonged to R-DC-like cells. The fourth column indicates whether the scRNA-seq and scATAC-seq profiles came from the same cell, meaning scPairing recapitulated the true pairing of the two modalities. The cell barcodes listed have had their prefixes stripped off, but no duplicate barcodes are present in the R-DC-like cells.

	RNA-ATAC	RNA-epitope	ATAC-epitope
Mean Cosine Similarity	0.9579	0.9060	0.9253
Median Cosine Similarity	0.9640	0.9183	0.9366
FOSCTTM	0.05378	0.1620	0.1294

Table S6: Mean and median pairwise modality cosine similarities, and FOSCTTM between the true matching triplets from DOGMA-seq, related to Fig. 7. The cosine similarities and FOSCTTM are calculated using the embeddings produced by applying scPairing with TEA-seq as the bridge data. The similarity and FOSCTTM is computed between each pair of modalities.

References

1. Zhu, K., Bendl, J., Rahman, S., Vicari, J.M., Coleman, C., Clarence, T., Latouche, O., Tsankova, N.M., Li, A., Brennand, K.J. et al. (2023). Multi-omic profiling of the developing human cerebral cortex at the single-cell level. Science Advances *9*, eadg3754. doi: https://doi.org/10.1126/sciadv.adg3754.

2. Wang, S.K., Nair, S., Li, R., Kraft, K., Pampari, A., Patel, A., Kang, J.B., Luong, C., Kundaje, A., and Chang, H.Y. (2022). Single-cell multiome of the human retina and deep learning nominate causal variants in complex eye diseases. Cell Genomics *2*, 100164. doi: https://doi.org/10.1016/j.xgen.2022.100164.

3. Hu, J., Wang, S.G., Hou, Y., Chen, Z., Liu, L., Li, R., Li, N., Zhou, L., Yang, Y., Wang, L. et al. (2024). Multi-omic profiling of clear cell renal cell carcinoma identifies metabolic reprogramming associated with disease progression. Nature Genetics *56*, 442–457. doi: https://doi.org/10.1038/s41588-024-01662-5.

4. Single Cell Immune Profiling Dataset by Cell Ranger ARC 2.0.0, 10x Genomics (2021). 10k Human PBMCs, Multiome v1.0, Chromium X. .

5. Yu, Z., Lv, Y., Su, C., Lu, W., Zhang, R., Li, J., Guo, B., Yan, H., Liu, D., Yang, Z. et al. (2023). Integrative single-cell analysis reveals transcriptional and epigenetic regulatory features of clear cell renal cell carcinoma. Cancer Research *83*, 700–719. doi: https://doi.org/10.1158/0008-5472.CAN-22-2224.
